# Quantitative Effects of Anthropogenic and Natural Factors on Heavy Metals Pollution and Spatial Distribution in Surface Drinking Water Sources in the Upper Huaihe River Basin in China

**DOI:** 10.3390/toxics12070517

**Published:** 2024-07-18

**Authors:** Tong Liu, Mingya Wang, Chunhui Zhang, Shili Yang, Fan Zhang, Luhao Jia, Wanqi Ma, Shaobo Sui, Qingwei Liu, Mingshi Wang

**Affiliations:** College of Resource and Environment, Henan Polytechnic University, Jiaozuo 454003, China; 212203020060@home.hpu.edu.cn (T.L.); wangmy@hpu.edu.cn (M.W.); mwq15931158185@outlook.com (W.M.); liuqingwei815@126.com (Q.L.)

**Keywords:** drinking water source area, Huaihe River Basin, heavy metals, geodetector

## Abstract

The water quality of sources in the Huaihe River Basin significantly affects the lives and health of approximately 16.7% of China’s population. Identifying and quantifying pollution sources and risks is essential for effective water resource management. This study utilized Monte Carlo simulations and Geodetector to assess water quality and eutrophication, as well as to evaluate the sources of heavy metals and the associated health risks for both adults and children. The results showed that eutrophication of water sources in Huaihe River was severe, with an overall EI value of 37.92; 67.8% of the water sources were classified as mesotrophic and 32.2% classified as eutrophic. Water quality and eutrophication levels in the southern mountainous regions were better than those in the densely populated northern areas. Adults were found to have a higher carcinogenic risk than children, whereas children faced a higher noncarcinogenic risk than adults. Cr presented the highest carcinogenic risk, affecting more than 99.8% of both adults and children at levels above 1 × 10^−6^ but not exceeding 1 × 10^−4^. The noncarcinogenic risk from metals did not surpass a level of 1, except for Pb. As was primarily influenced by agricultural activities and transportation, whereas Cd, Cr, and Pb were mainly affected by industrial activities, particularly in local textile industries such as knitting and clothing manufacturing. The analysis demonstrated that the influence of anthropogenic factors on heavy metal distribution was significantly enhanced by indirect natural factors. For example, the explanatory power of Precipitation and Road Network Density on As was 0.362 and 0.189, respectively, whereas their interaction had an explanatory power as high as 0.673. This study indicates that the geodetector method is effective in elucidating the factors influencing heavy metal distribution in water, thereby providing valuable insights into pollution sources in global drinking water.

## 1. Introduction

Drinking water sources play a critical role in human health, as they are major carriers of drinking water resources [1,2]. The World Health Organization has reported that drinking water pollution accounts for 80% [3] of human diseases in more than 5 million deaths annually [4]. Heavy metals are one of the major contributors to poor drinking water quality. Surface drinking water sources are particularly vulnerable to human activities due to their low flow rates and long static residence times [5], making them unstable and fragile [1]. Despite the safety and security of drinking water sources being highly valued, rapid urbanization and industrialization have led to significant pollution in environmental water bodies worldwide [6], resulting in issues such as eutrophication and heavy metal contamination in regions where drinking water sources are located [7,8].

Eutrophication of water bodies is caused by excessive nitrogen, phosphorus, and other nutrients entering natural water bodies, leading to deterioration of water quality. Higher concentrations of nitrogen can exacerbate aquatic biological diseases [9], posing a risk to human health through the food chain. On the other hand, heavy metals present in drinking water can cause neurological, cardiovascular, visceral, and bone diseases [10,11] and, in severe cases, even death. Adults and children face varying degrees of threats from heavy metal exposure due to their physiological characteristics and individual differences [12]. Therefore, we introduce the Monte Carlo uncertainty analysis model to assess the differences in health risks of heavy metals among different age groups [13].

It is crucial to identify and quantify the sources of heavy metal pollution in order to prevent the harm to human health from heavy metals. The sources of heavy metals in water are complex, with contributions from both natural and anthropogenic sources. The accumulation of trace elements in water is a result of direct and indirect processes [14,15]. Multivariate statistical analysis, including correlation analysis, principal component analysis, and positive matrix factorization (PMF), is commonly used in studying the correlation between pollution severity and pollutant sources [16,17]. These methods are data-driven and do not require explicit information on emission factor propagation processes and source composition [18]. However, these studies often overlook the association between the spatial distribution of heavy metals and environmental factors.

In contrast, the Geodetector statistical method is not affected by linearity or collinearity from multiple variables [19]. It can test both the stratified heterogeneity of a single variable and the coupling of the spatial distribution of two variables to explore possible causal relationships between them and autonomously differentiate between natural and anthropogenic factors, quantifying the impact of their interactions on heavy metal contamination [20]. Geodetectors have been widely used in various environmental studies, including studies on soils [21], ecological vulnerability [22], and heavy metals [23], but their application to source apportionment of heavy metals in water is limited. This is mainly due to the unsuitability of the model for individual rivers and lakes with narrow widths, small spatial spans, and low spatial heterogeneity. However, in this study, the drinking water source areas are widely distributed across the study area, exhibiting significant spatial heterogeneity. Therefore, geodetectors can be applied to water source areas in this research.

The Huaihe River Basin, one of the most densely populated areas in China [24], and the quality of its water sources affects the lives and health of approximately 16.7% of the country’s population [25]. The study area consists of 98.2% of the upper Huaihe River Basin [26], which is responsible for transporting nearly 10 billion cubic meters of water annually to the middle and lower reaches [25]. Since 2008, the level of urbanization in the upper basin has experienced exponential growth [27,28]. Due to dense population, high urbanization, and clustered factories [29], there are some eutrophication [30] and heavy metal pollution problems in the drinking water sources of the Huaihe River Basin [31]. Direct discharges of sewage and hazardous waste are prohibited in protected areas of drinking water sources [32]; therefore, indirect pollution impacts from anthropogenic discharge may play a significant role. This study incorporates precipitation and DEM, which are natural factors heavily influenced by human activities, into the selection of geodetector factors. This study intends to reveal the indirect influences of man-made factors on drinking water sources through the interaction between these natural factors and anthropogenic factors.

Based on the aforementioned discussion, the objectives of this study are as follows: (1) Analyze the water quality, eutrophication status, and spatial distribution of drinking water sources in the upper reaches of the Huaihe River Basin. (2) The carcinogenic and noncarcinogenic risks posed by five heavy metals to adults and children were evaluated using the Monte Carlo model. (3) The influence of each factor on heavy metals was quantified, and the main sources of heavy metal contamination were identified. The findings of this study indicate that the geodetector method is a useful approach for understanding the factors influencing the distribution patterns of heavy metals in water. Additionally, it can provide universally applicable results regarding pollution sources for drinking water sources worldwide, offering valuable information for the prevention and control of water pollution.

## 2. Materials and Methods

### 2.1. Study Area

**Figure 1:**
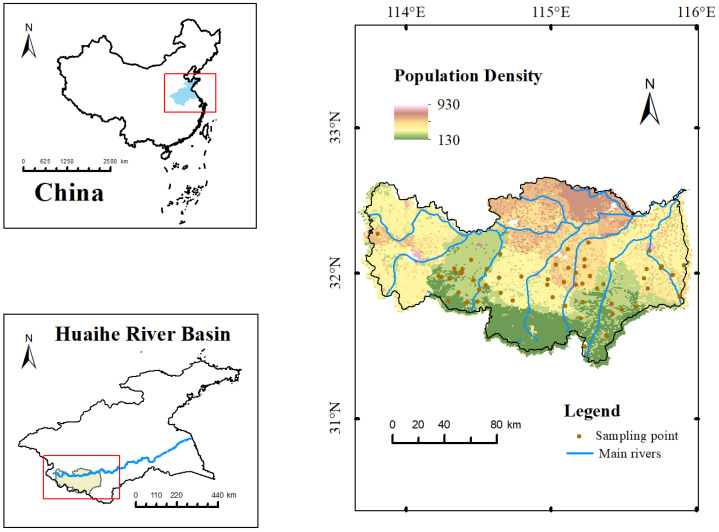
Sampling points in the research area. (The blue part in the figure represents the Huai River Basin, the yellow part represents the study area, and the blue line represents the Huai River).

The Huai River originates from the Tongbai Mountain area in Henan Province and flows in a west-to-east direction. The Huaihe River Basin serves as a transitional zone between the climates of northern and southern China. The northern part of the basin experiences a warm temperate semihumid monsoon climate, whereas the southern part has a subtropical humid monsoon climate [33]. The research area is located to the south of the Huai River and represents the southernmost point of Henan Province. It lies in the middle and upper reaches of the Huai River, bordering Hubei and Anhui provinces, and is considered a border area among the three provinces. The terrain is characterized by a north-to-south terraced pattern, with higher elevations in the south and lower elevations in the north. The western and southern parts consist of Tongbai Mountain and Dabie Mountain, forming the Yunan Mountains. Rivers flow into the Huai River from south to north [34]. The research area map is shown in Figure 1.

### 2.2. Data Sources

This study utilized monitoring data collected between January and October 2021 from 59 sampling points within the upper Huaihe River Basin. The sampling points included 39 lake-type drinking water sources and 20 nonlake-type drinking water sources. The daily water supply range of the selected drinking water source for sampling points is from 800 to 20,000 tons, serving a population of 5000 to 22,253 people. It covers various regions within the scope of this study, representing various types of water sources. Each sampling point provided data from the wet season, dry season, and normal season, totaling 236 data points. The flood season of the Huai River is usually from June to August, the dry season is from December to February, and the rest of the months constitute the normal water period [35]. In 2021, a heavy rainstorm event occurred in Henan, where the study area is located, and the rainfall reached the highest level in the past decade. However, in the analysis of this study, it was found that there was no significant difference in water quality before and after heavy rain weather, so sampling and data analysis were only conducted in 2021. This study takes into account this factor and samples were taken at different time periods. The data collected from July to August are classified as the wet season, while the data from January to March are classified as the dry season. The data from April to May and October are classified as normal season data. The data included various parameters, including pH, DO, COD_Mn_, BOD_5_, NH3-N, TP, TN, As, Hg, Cd, Cr^6+^, Pb, and NO_3_-N. The data were sourced from the Henan Provincial Environmental Testing Center.

### 2.3. Sample Collection and Analysis

Surface water samples were collected and stored in 1.5 L high-density polyethylene plastic bottles. Prior to sample collection, the bottles were thoroughly cleaned and rinsed with the water samples to ensure the absence of air bubbles. The flowing water was collected at a depth of approximately 0.5 m, depending on site conditions. Water samples intended for metal content analysis were field-filtered using a 0.22-μm aqueous filter membrane and subsequently stored in 50-mL polypropylene centrifuge tubes. Two drops of nitric acid were added to prevent metal reactions (pH < 2). Additionally, pH and DO measurements were conducted on-site using instruments with accuracies of 0.01 pH units and 0.01 mg/L, respectively. Coordinate information was recorded using a global positioning system (GPS) device.

After collection, the samples were sealed to protect them from light and transported to the laboratory for storage at temperatures below 4 °C. To ensure accuracy, the experiments were conducted within 48 h of sample collection. The experimental methods followed the Environmental Quality Standards for Surface Water (GB3838-2002) [36]. For the measurement of COD_Mn_, the dichromate method was used. The degradation of organic wastewater by microorganisms was used to determine the BOD_5_ concentration by appropriately diluting the water sample and inoculating it with a culture medium containing active microorganisms. The concentrations of NH3-N, TP, TN, and NO3-N were measured using a spectrophotometer. The metals in the water samples were directly tested using an inductively coupled plasma mass spectrometer (ICP-MS; NexIon 350, PerkinElmer, Waltham, MA, USA) for As, Cd, Cr, and Pb and by atomic fluorescence (atomic fluorescence photometer AFS-8220 model, Engineering Center Analytical Instrument, Beijing Jitian Instrument Co., Ltd., Beijing, China) for Hg determination.

### 2.4. Quality Assessment and Quality Control

To ensure the accuracy of the results, quality assurance and quality control measures were implemented throughout the experimental process. This included the use of a multielement standard solution (GNM-M242887-2013) at a concentration of 100 μg/mL (from China Nonferrous Metals Institute), an Hg standard solution (GSB 07-1274-2000) at a concentration of 100 mg/L, and an As standard solution (GSB 07-1275-2000) at a concentration of 100 mg/L (from the Standard Sample Research Institute of the Chinese Ministry of Environmental Protection). The standard recovery rate was maintained between 90% and 105%. Calibration curves were constructed using a series of solutions to calculate the concentration of the samples. Each batch of experimental samples included 20% blank samples and 10% parallel samples. The blank samples had detection levels below the limit, and the relative deviation of the determinations was controlled within ±5%, meeting the experimental requirements. Some of the raw data are presented in Table S5.

### 2.5. Research Methods

#### 2.5.1. Water Quality Index (WQI)

The WQI is a widely used tool for assessing the quality of surface water and groundwater [37]. It combines the monitoring results of several water quality parameters to provide a comprehensive analysis, reflecting the overall situation of the water body [38]. In this study, the WQI was calculated using the following formula for eight water quality parameters: pH, DO, COD_Mn_, BOD_5_, NH_3_-N, TP, TN, and NO_3_-N [39]:

(1) $$WQI=k\sum_{i}^{n}C_{i}P_{i}/\sum_{i}^{n}P_{i}$$

In the equation, the constant *k* is a subjective factor with a numerical range of 0.25–1, consisting of four levels: 1.00 represents no significant water pollution, 0.75 indicates slight pollution, 0.50 indicates obvious pollution, and 0.25 indicates high pollution. This study analyses the quality of drinking water sources and selects a value of 1.00 for the constant *k*. *n* represents the number of water quality parameters, *C_i_* represents the normalized value assigned to parameter *i*, and *P_i_* represents the weight assigned to parameter *i*. The values of *P_i_* and *C_i_* are based on studies by Sevgili et al. [40] and Zhu et al. [41], and the range of *P_i_* is between 1–4. In this study, the parameters TP and TN had relatively high pollution levels, so their weights were adjusted to 4. The standards for water quality classification are shown in Table S1.

#### 2.5.2. Universal Index Formula in the Form of a Logarithmic Power Function

The universal index formula proposed by Li et al., which takes the form of a logarithmic power function, is applicable for assessing eutrophication in freshwater bodies in China [42,43]. In this study, seven water quality indicators, namely, DO, TP, TN, COD_Mn_, BOD_5_, NH_3_-N, and NO_3_-N, were calculated using the logarithmic power function universal index formula for assessing nutritional status:

(2) $$EI_{j}=10.77\times {\left(\ln x_{j}\right)}^{1.1826}$$

(3) $$EI=\sum_{j}^{n}W_{j}\times EI_{j}$$

*x_j_* is the normalized value of index *j*; *C_j_* is the measured value of index *j*; *C_j_*_0_ selects the “extremely poor” nutritional value of the index, and its reference is Li et al. [43]; *W_j_* is the normalized weight value of index *j*, and each index can be regarded as an equal weight, so *W_j_* = 1/*n* [44]; and n is the number of selected evaluations. By comparing the EI value with Table S2, the eutrophication assessment grade of the water body can be obtained.

#### 2.5.3. Health Assessment

HRA is a method that quantitatively associates environmental pollution with human health, evaluating the health risks associated with exposure to the environment [45,46]. This study adopts the health risk assessment model recommended by the US Environmental Protection Agency (USEPA), which includes both carcinogenic and noncarcinogenic risk assessments. Each factor poses a threat to human health, mainly through oral intake, skin contact, and respiratory intake [47]. For drinking water, the main exposure route for humans is oral intake, so this study only considered oral intake. The daily exposure measurement formula for the ADD is as follows:

(4) $$ADD=\frac{C\times IR\times EF\times ED}{BW\times AT}$$

In the formula, *ADD* is the daily average exposure level (mg/(kg·d)); *C* is the measured value of the element (mg/L); *IR* is the daily average oral intake (L/d); *EF* is the annual exposure frequency (d/a); *ED* is the exposure cycle (a); *BW* is the body weight of the human body (kg); and *AT* is the average time of exposure (d).

The formulas for the carcinogenic and noncarcinogenic risks of various factors for different populations are as follows [48]:

(5) Carcinogenic: CR=ADD×SF

(6) Noncarcinogenic: HQ=ADD/RfD

*SF* represents the reference dose for carcinogenic elements in this exposure pathway (mg/(kg·d)); *RfD* represents the reference dose for noncarcinogenic elements in the exposure pathway of this element (mg/(kg·d)). A *CR* value less than 1.0 × 10^−6^ indicates a lower risk of carcinogenesis, whereas a *CR* value between 1.0 × 10^−6^ and 1.0 × 10^−4^ indicates a certain risk of carcinogenesis. A *CR* > 1.0 × 10^−4^ indicates a high risk of cancer and should receive special attention. An *HQ* value ≤ 1 indicates that the exposure level does not surpass the threshold for adverse reactions, resulting in a low noncarcinogenic risk. An *HQ* greater than 1 indicates that the exposure level exceeds the threshold, indicating a high noncarcinogenic risk. The values of *SF* and *RfD* for each factor can be found in Table S3 [49].

#### 2.5.4. Monte Carlo Simulation (MCS)

Monte Carlo simulation is a widely used mathematical model in health risk assessment [50] that utilizes probability and statistical mathematical theory to achieve greater accuracy in uncertainty analysis [51]. This study evaluated the carcinogenic and noncarcinogenic risks of As, Hg, Cd, Cr^6+^, and Pb in children and adults. A total of 10,000 random simulations were conducted for both the child and adult groups to obtain relatively stable results. The various factors in the ADD calculation formula refer to the USEPA and other studies [51,52,53,54], as shown in Table S4.

#### 2.5.5. Geodetector

Geodetectors are based on spatial distribution theory and employ spatial statistical methods to detect and quantify the level of influence between independent and dependent variables. In recent years, it has been widely applied in ecosystem analysis. This study mainly utilizes factor detection and interactive detection in geographical detectors to accomplish single factor detection and factor interactive detection, respectively [19].

(1) Factor detection

Factor detection is utilized to identify the explanatory power of the driving factor causing spatial differentiation in the dependent variable. The formula is as follows:

(7) $$q=1-\frac{1}{N\sigma^{2}}f\left(x\right)\sum_{h=1}^{L}N_{h}\sigma_{h}^{2}$$

where *q* is the explanatory power of the factor to the dependent variable; *h* = 1, 2, …, *L*; *L* is the grade or classification of the dependent variable and different independent variables; *N_h_* and *N* are the number of samples in different grades of regions and the whole region, respectively; and $\sigma_{h}^{2}$ and *σ*^2^ are the variance of dependent variables in different grades of regions and the whole region, respectively. The larger the *q* value is, the greater the impact of this factor on the dependent variable.

(2) Interaction detector

The explanatory power of the interaction between independent variables on the dependent variable is determined by identifying the q-value when two different independent variables interact. This interaction is categorized into five categories: bivariable enhancement: q(X1∩X2) > max[q(X1),q(X2)], nonlinear enhancement: q(X1∩X2) > q(X1) + q(X2), independent: q(X1∩X2) = q(X1) + q(X2), nonlinear weakening: q(X1∩X2) < min[q(X1),q(X2)], and unidirectional weakening: min[q(X1),q(X2)] < q(X1∩X2) < max[q(X1),q(X2)].

Referring to the factor indicator selection methods of other scholars, combined with the sources of heavy metals in soil and considering the difficulty of data collection and the actual situation of the research area, nine influencing factors were selected: DEM, NDVI, Precipitation, Temperature, Soil Type, Land Use, Road Network Density, GDP, and Population Density [55,56].

The Precipitation and Temperature image and vector data were obtained from the National Earth System Science Data Center. The Road Network Density data come from the National Catalogue Service for Geographic Information. All other data were obtained from the Resource and Environment Science and Data Center.

#### 2.5.6. Data Analysis

The software used in this study included Excel 2022, ArcMap 10.5, Origin 2022, and Oracle Crystal Ball 11.1.3.0.0.

## 3. Results and Discussion

The descriptive statistical results of the concentrations of various indicators in the drinking water source area are shown in Table S5. According to the “Environmental Quality Standards for Surface Water” (GB3838-2002) [36], the water function and classification standards stipulate that centralized drinking water sources should at least meet the water quality requirements of Class III water bodies. 

After comparing the concentration detection results with the Class III standard, it was found that the TP exceeded the standard by 0.4%, the TN exceeded the standard by 2.5%, and all other indicators met the Class III water standard without exceeding the standard. The coefficient of variation between Cr^6+^ and Pb exceeded 90%, indicating that the concentrations of Cr^6+^ and Pb were relatively high in certain areas of the drinking water source in the study area. There are significant spatial differences in Cr^6+^ and Pb in different water sources. The high coefficient of variation may be due to specific point source emissions or the influence of the surrounding geological environment.

### 3.1. Assessment of Water Quality Pollution in Drinking Water Source Areas

#### 3.1.1. Comprehensive Index Evaluation of Water Quality

**Figure 2:**
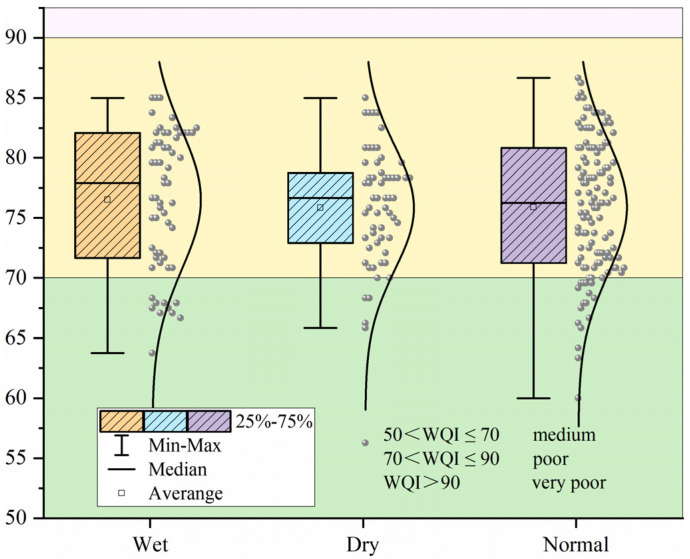
WQI water quality index during wet, dry, and normal seasons.

The WQI index method, which utilizes various weights and indicators for comprehensive analysis and evaluation, is employed to assess the overall water quality of drinking water sources. The WQI remains relatively consistent during the wet season, dry season, and normal season, with similar mean values. Additionally, the median value during the wet season slightly surpassed that of the other two periods (Figure 2). Based on the calculation data, the comprehensive evaluation of the water bodies at point 59 revealed that 52 points exhibited good water quality, whereas 7 points exhibited medium water quality. Among the 52 points with a good comprehensive water quality evaluation, 38 points consistently demonstrated good water quality across all three periods, whereas 14 points exhibited medium water quality in a specific period. These variations occur sporadically and are not subject to any clear pattern. These results underscore the generally positive water quality of drinking water sources in the upper reaches of the Huaihe River Basin. Based on the aforementioned analysis, 35.6% of the water quality assessment points in a specific period are classified as medium. Consequently, it is crucial to continuously monitor the water quality safety of drinking water in the corresponding areas.

#### 3.1.2. Water Eutrophication Assessment

In this study, a comprehensive evaluation of 59 sampling points was conducted to assess the nutritional status of water bodies using the EI value. The findings revealed that 40 points had a mesotrophic grade 2 water quality nutrition status, whereas 19 points had a eutrophic grade 3 water quality nutrition status. It is important to note that the water bodies at sampling points that are still in the intermediate trophic level exhibit EI values that are mostly above 30, nearing the eutrophication standard of 39.42 for the third grade. Consequently, the water bodies in these areas are at risk of worsening eutrophication due to human activities. 

To comprehend the spatial characteristics of water quality in drinking water sources within the upper reaches of the Huaihe River Basin, this study conducted a visual analysis utilizing the results of water quality evaluation indices from different periods and eutrophication status through the kriging interpolation method (Figures S1 and S2). The results showed similar characteristics between the two. The spatial distribution patterns of water quality during the wet season and dry season are similar, with the southwest and southeast regions displaying significantly better water quality than the other regions. Moreover, the water quality in the western and southern regions was greater than that in the eastern region. The eutrophication status aligns with the comprehensive index evaluation of water quality; the regions in the southwest and southeast exhibit lower eutrophication degrees and better water quality. A possible reason is that the terrain of the study area has higher elevations in the south and lower elevations in the north, with rivers flowing from south to north into the Huaihe River. The southwest region, which serves as the birthplace of the river or the upstream region, exhibits superior water quality. As water flows from mountainous areas with minimal human activity to plains areas with increased human activity, water quality gradually deteriorates, and eutrophication levels increase. These findings highlight the significant impact of human activities on water bodies. Although China places significant emphasis on protecting centralized drinking water sources compared to other surface water resources, the lowest level of eutrophication for water bodies in this study remains at the mesotrophic level. In fact, 32.2% of the sampling points in the study fall within the third grade of eutrophication. 

**Figure 3:**
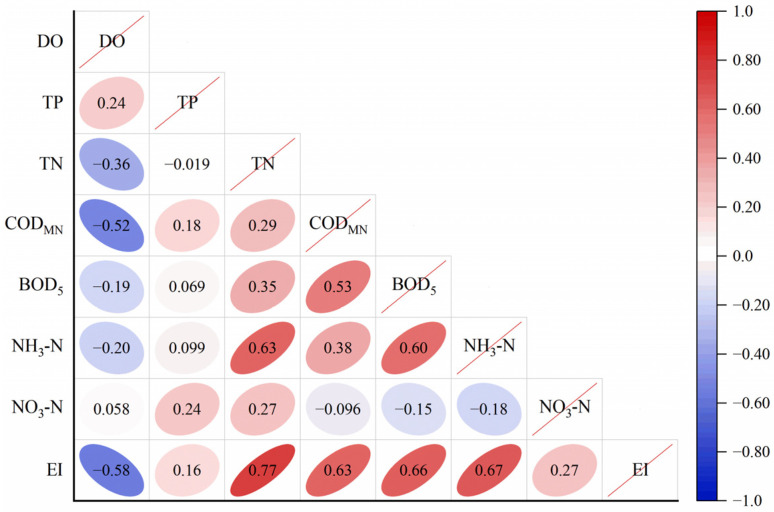
Correlation analysis between EI and various factors.

To gain a better understanding of the water quality parameters that influence the degree of eutrophication (EI), a Spearman correlation analysis was conducted between the indicators of drinking water sources in the upper reaches of the Huaihe River Basin and the EI. The results indicate that the main parameters impacting EI include DO, TN, COD_Mn_, BOD_5_, and NH_3_-N (Figure 3). It was positively correlated with TN (0.77), COD_Mn_ (0.63), BOD_5_ (0.64), and NH_3_-N (0.64) showed positive correlations with EI, whereas DO (−0.57) showed a negative correlation. Notably, TN, which has the most significant impact on water eutrophication, also exceeded the standard in this study. 

Given that the study area is a significant agricultural production zone, excessive nitrogen and phosphorus levels, as well as inefficient utilization rates of chemical fertilizers [57], likely contribute to eutrophication in water bodies [58]. This result can be attributed to the study by Zhang et al. [59], which highlighted the impact of excessive chemical fertilizer application on China’s water environment, identifying chemical fertilizers as the main contributors to nitrogen and phosphorus levels in surface water. According to the Henan Provincial Statistical Yearbook, the study area consumed 200,299 tons of nitrogen fertilizer and 70,601 tons of phosphorus fertilizer in 2021. Peng et al. [60]. Found, in their study of the Laohutan Reservoir in the Yangtze River Basin, that the reservoir is in a moderately eutrophic state, and its source of ammonia nitrogen may come from the excretion of fish such as silver carp and bighead carp, the release of ammonia nitrogen from sediment, and the decomposition of endogenous organic nitrogen. Dan et al. [61]. Found, in their research on the Sanmenxia reservoir area of the Yellow River, that the natural river channel section of the reservoir is nutrient poor and generally moderately nutrient deficient, mainly due to the local green algae and diatom phytoplankton. In total, 68% of the 71 large lakes worldwide show an increasing trend of shallow eutrophication intensity in summer [62]. Zhu et al. [63]. conducted a survey of 22 representative urban reservoirs and found that the total nitrogen of most reservoirs had reached the IV level, while the total phosphorus was mostly at the III level. Eutrophication of lakes and reservoirs is a global trend, and the reasons for eutrophication may vary in each region. The specific reasons for eutrophication in different regions are the key to controlling local eutrophication problems. Controlling the excessive use of chemical fertilizers in agricultural activities in this study area may improve the problem of eutrophication in water bodies. Although the level of middle nutrition is still within an acceptable range, there remains a need for continued efforts to address eutrophication levels and protect water bodies to safeguard drinking water sources in the region.

### 3.2. Health Risk Assessment of Heavy Metals in Drinking Water Source Areas

**Figure 4:**
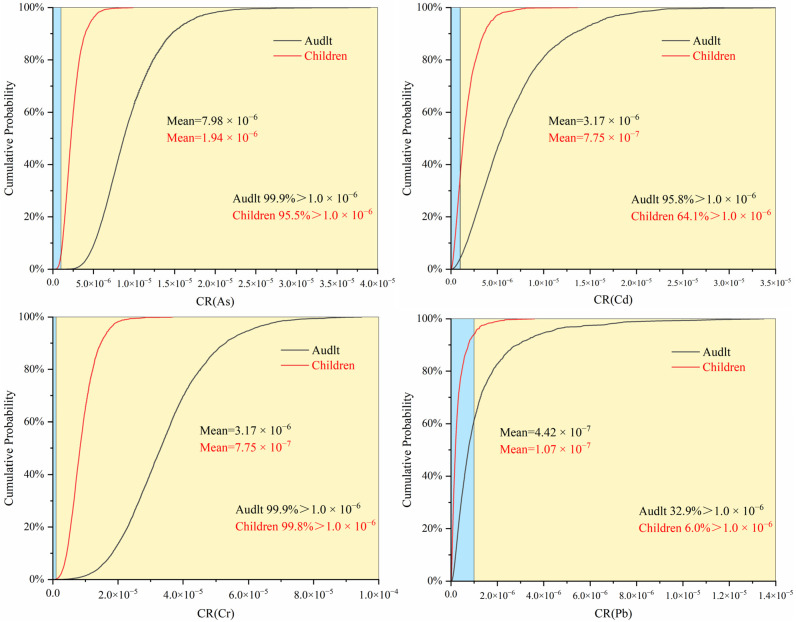
Frequency chart of cumulative carcinogenic risks for various heavy metals.

A Monte Carlo simulation method was used to assess the carcinogenic and noncarcinogenic risks posed by heavy metals to adults and children. The health risk table and cumulative frequency chart constructed based on these simulations reveal differences in the carcinogenic risk of heavy metals between children and adults. The specific calculation results, including average and average standard error, are presented in Tables S6 and S7. The findings indicate that both populations face cancer risks, but the risks are greater in adults than in children. The maximum carcinogenic risk observed for all populations remained below the threshold (1 × 10^−4^). However, the carcinogenic risks of As and Cr^6+^ in children and adults, as well as the average carcinogenic risk of Cd in adults, exceed the limit set by the US Environmental Protection Agency (1 × 10^−6^), indicating a potential carcinogenic risk. Additionally, as shown in Figure 4, that the overall cancer risk for both populations was relatively low. However, a considerable number of individuals are exposed to low cancer risks from As and Cr^6+^, as indicated by the cumulative frequency exceeding 90%. The order of carcinogenic risk from high to low was As > Cr^6+^ > Cd > Pb. The greater risk for adults than for children can be attributed to longer periods of pollutant exposure as individuals age, resulting in a greater cancer risk for adults. Feng et al.’s [31] study on drinking water in the Huaihe River Basin from 2015 to 2019 has similar conclusions to this study, with As and Cr^6+^ having the highest carcinogenic risk.

**Figure 5:**
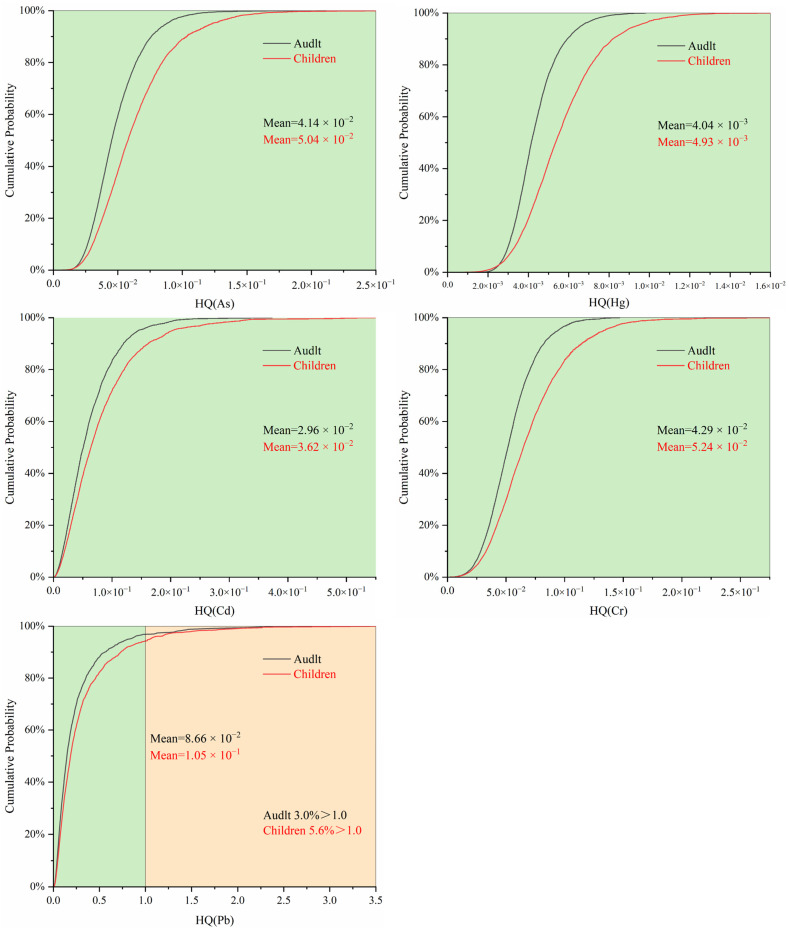
Frequency chart of cumulative noncarcinogenic risks for various heavy metals.

For noncarcinogenic risks, children are at greater risk and have higher cumulative frequencies than adults. The average noncarcinogenic risk for both populations did not exceed the threshold of 1, indicating a lower noncarcinogenic risk overall. The only maximum noncarcinogenic risk for both populations was for Pb, which exceeded 1. Figure 5 shows that exposure to Pb poses a greater noncarcinogenic risk for 3.0% of adults and 5.6% of children. The order of noncarcinogenic risk from high to low was Pb > Cd > Cr^6+^ > As > Hg. This is similar to the conclusion drawn by Chen et al. [64] on the groundwater and soil of villages in the Huai River Basin passing through Henan Province, where Pb and Cr are the main noncarcinogenic risk factors. However, none of their studies mentioned the source analysis of heavy metals. To investigate the pollution sources of heavy metals and the pollution patterns in the Huai River Basin, this study carried out follow-up work.

Children are at greater risk than adults because of their incomplete physiological development and heightened sensitivity to heavy metal toxicity. As individuals age, their intake per unit weight gradually decreases, resulting in a lower noncarcinogenic risk for adults than for children. Research has also demonstrated differences in carcinogenic and noncarcinogenic risks between adults and children. Although the adult population is more vulnerable to the carcinogenic health risks associated with heavy metal exposure, children have a greater risk value for noncarcinogenic risks than adults. These disparities primarily stem from variations in physiological characteristics and age. Consequently, both children and adults should receive attention and protection through strategies and policies aimed at controlling and reducing heavy metal exposure. To better safeguard public health, corresponding measures should be taken to reduce the emission and exposure risks of heavy metal pollutants, and monitoring and evaluation efforts should be strengthened. This will ensure a safe and sustainable environment for everyone. The research results presented here hold significant reference value for formulating and implementing environmental protection policies, as well as managing the health of children and adults.

### 3.3. Source Apportionment of Heavy Metals in Drinking Water Sources

#### 3.3.1. Factor Detection

**Figure 6:**
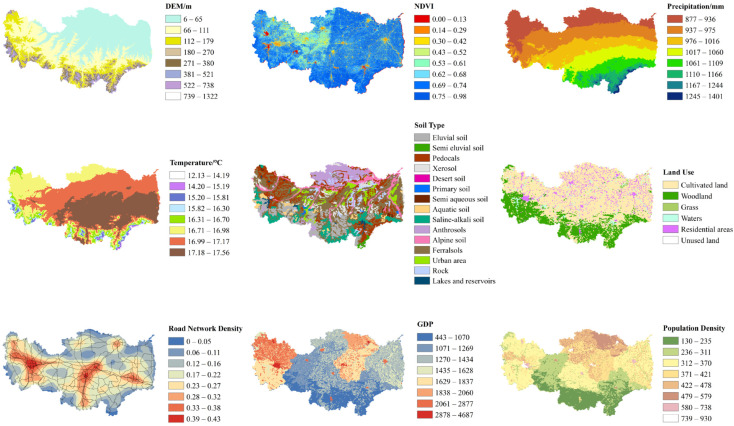
Classification of influencing factors.

The selection of factors referred to the selection methods of other scholars combined with the sources of heavy metals in water and the actual situation of the research area. Five natural factors and four anthropogenic factor were selected: DEM, NDVI, Precipitation, Temperature, Soil Type and Land Use, Road Network Density, GDP, and Population Density, respectively. Their spatial distribution map is shown in Figure 6. To analyze the pollution sources of heavy metals in the upper reaches of the Huaihe River Basin, quantitative analysis was conducted via principal component analysis and correlation analysis (Figure S3). The results showed negative correlations between As and Cd (−0.57) and Pb (−0.52), whereas Cd had positive correlations with Cr (0.70) and Pb (0.93), and Cr had a positive correlation with Pb (0.74). This suggests that these heavy metals may have similar sources or be influenced by similar factors. No significant correlation was found between Hg and the other heavy metals. Principal component analysis revealed two principal components with a cumulative contribution rate of 80.916% through orthogonal rotation. The first group, with a contribution rate of 58.013%, was characterized by high loadings of As, Cr, Cd, and Pb, with values of 0.830, 0.842, 0.939, and 0.775, respectively, indicating consistent sources for these heavy metals. The second group, with a contribution rate of 22.903%, had a high loading of Hg (0.947), suggesting that the source of Hg may differ from that of other heavy metals.

**Figure 7:**
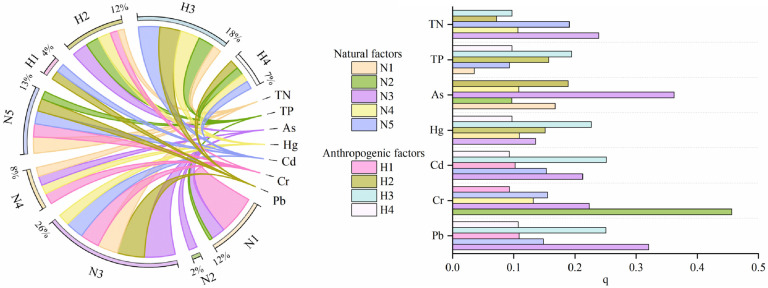
The explanatory power *q* value of influencing factors on TN, TP, and heavy metals (N_1–5_ represents DEM, NDVI, Precipitation, Temperature, Soil Type, H_1–4_ represents Land Use, Road Network Density, GDP, Population Density).

Figure 7 presents the explanatory power q values of nine factors for TN, TP, and five heavy metals. Although there were differences in the explanatory power of different factors for TN, TP, and heavy metals, overall, factors such as DEM, Precipitation, Soil Type, Road Network Density, and GDP exhibited strong explanatory power for the spatial distribution of each element. The main influencing factors for TN were Precipitation (0.239) > Soil Type (0.191) > Temperature (0.107) and GDP (0.097). The main influencing factors for TP were GDP (0.194) > Road Network Density (0.157) > Population Density (0.097). The main influencing factors for As were Precipitation (0.362) > Road Network Density (0.189) > DEM (0.168). The main influencing factors for Hg were GDP (0.227) > Road Network Density (0.151) > Precipitation (0.136). The main influencing factors for Cd were GDP (0.251) >Precipitation (0.213) > Soil Type (0.153). The main influencing factors for Cr were DEM (0.457) > Precipitation (0.223) > Soil Type (0.155). The main influencing factors for Pb were Precipitation (0.321) > GDP (0.251) > Soil Type (0.148). 

#### 3.3.2. Interaction Detection

The composition of water resources in drinking water sources is complex, and the spatial distribution of heavy metal pollution is not solely determined by a single factor. It is often the result of the combined influence of multiple natural and human factors. Interactive detection is beneficial for accurately determining the underlying driving mechanisms that affect the spatial distribution of heavy metals [65]. The explanatory power of the interaction between any two factors on the spatial differentiation of TN, TP, and the five heavy metals was greater than that of a single factor, with most interactions exhibiting nonlinear enhancement and a few showing bivariable enhancement (Table S8). Factors with dominant single-factor explanatory power generally exhibited greater explanatory power in their interactions.

The diverse influences of various factors on different heavy metals highlight the heterogeneous mechanisms behind heavy metal changes. The analysis of the factor detector and interaction detector results revealed significant differences in the single-factor influences on TN and TP in the study area. Natural factors such as Precipitation and Soil Type are the primary factors influencing TN, whereas TP is influenced mainly by human factors such as GDP, Road Network Density, and Population Density. The correlation analysis indicated no significant correlation between TN and TP, suggesting that there were different sources for these two metals. The findings from the interaction detection demonstrate that the main factors impacting TN exhibit nonlinear enhancement when interacting with human factors such as Population Density and GDP. The greater consumption of nitrogen fertilizer than of phosphorus fertilizer suggests that TN primarily accumulates in rivers and lakes through the interaction between fertilizer application and natural factors and is ultimately carried by precipitation. Moreover, TP sources may include domestic sewage and industrial activities. Even without direct human emissions to water sources, pollutants can still affect the safety of drinking water quality indirectly through natural factors such as precipitation. Therefore, in addition to controlling the use of fertilizers in agricultural activities, it is also necessary to control pollution emissions from factories, urban life, and other sources, and stay away from the surrounding areas of drinking water sources to protect the safety of drinking water quality.

The influencing factors for heavy metals can be divided into natural factors and human factors. The natural factors included Precipitation, DEM, and Soil Type, whereas the human factors mainly included GDP and Road Network Density. These factors are related to the terrain and industrial structure of the research area.

According to the Xinyang Statistical Yearbook, the economy of the study area in 2021 was dominated by agriculture and industry, with the primary and secondary industries accounting for 19.6% and 34.7% of the gross domestic product, respectively. In the southern and western regions of the research area, which are surrounded by mountains, agricultural and industrial activities produce waste gases, waste liquids, and residues containing heavy metals during production, processing, and transportation processes. Influenced by precipitation and elevation, these pollutants converge towards the soil, the Huai River, and central cities in the north through natural sedimentation, atmospheric precipitation, and surface runoff. They are mainly distributed around agricultural and industrial areas, as well as along highways and railways. The analysis of the interactive detection results confirmed the aforementioned findings. With the exception of Hg, the main influencing factors for other heavy metals are the interactions between human factors and natural factors. This interaction significantly improved the explanatory power of the heavy metals. For example, the contributions of Precipitation and Road Network Density as single factors are 0.362 and 0.189, respectively, whereas the explanatory power of their interaction is as high as 0.673.

The main factor affecting Hg is the interaction between two human factors, which is weakly explained by natural factors. The correlation analysis and principal component analysis mentioned earlier also indicate that the source of Hg differs from that of other heavy metals. High concentrations of Hg are found in the water source area around the urban center of Shangcheng County, Xinyang city, which has a dense population, factories, and developed transportation. Its surroundings include various sizes of factories, such as textile, doors and windows, electronics, etc. Considering the factors and interaction detection results of Hg, it is likely that human activities such as domestic sewage, industrial wastewater, and transportation all contribute to Hg contamination. In her study on heavy metal source apportionment at the county level, Yang et al. [66] found that 40% of As comes from natural and agricultural sources, whereas the remaining 60% comes from transportation and industrial emissions. The first three factors affecting As in the interactive detection results are the interactions between natural factors and road network density. The water source of the Zhaochong Reservoir type in Baidian Township, the Bailu River, which has a relatively high concentration of As, is located near a large area of arable land and is adjacent to the Shanghai Shaanxi Expressway. Therefore, the sources of As may be similar to those found in Yang Xue’s research. Wang et al. [67] reported that industrial waste residue, exhaust gas, and other sources are the main sources of Cd. In their research on pollutants in textile industry wastewater, He et al. [68] reported that almost all types of synthetic dyes in the textile industry contain Cd. Cr, Cd, and Pb are the most commonly found heavy metals in textile industry wastewater. GDP is the main factor affecting Cd, with two interaction detection results also involving GDP. The concentration of Cd is relatively high in the water source area of the Guanhe groundwater well in Majiji Township. After field investigation, it was found that there are multiple knitting factories, clothing factories, and clothing companies in the surrounding area. Therefore, the contamination of Cd may be primarily from such industrial emissions.

For the factor detection of Cr, the DEM is the main influencing factor, and the three factors affecting Cr in China according to the interaction detection results are interactions between the DEM and other factors. Precipitation is the main factor affecting Pb in factor detection, and the top three factors affecting Pb in China according to interaction detection are the interactions between precipitation and other factors. According to the correlation analysis, the correlation coefficient between Cr and Cd was 0.70, and the correlation coefficient between Pb and Cd was 0.93. Based on the spatial distribution study of heavy metal concentrations, it can be concluded that the sources of Cr, Pb, and Cd are similar. The water plant in Duanji township, which has a high concentration of Cr, has fewer factories near the water source area but is located at the intersection of the Huaigu Expressway and Shanghai Shaanxi Expressway. Therefore, the concentration of Cr may be affected by transportation, although the impact is not significant. The study by Li et al. [69] on the main stream of the Huai River Basin from 2014 to 2017 showed that natural factors such as flow rate and temperature are the main reasons affecting the distribution of heavy metals. When Ding et al. [70] analyzed the sources of heavy metals in sediments of the Jiangsu section of the Huai River, they found that the pollution sources may be a combination of urban sewage, industrial wastewater, and other pollutants. Based on the above analysis, compared with previous studies, the water quality of the Huai River has been continuously improving in recent years. Even though China explicitly prohibits direct discharge of pollutants into water sources, natural factors such as precipitation, elevation, and flow may carry pollutants into water sources. The areas with the most severe heavy metal pollution are usually in areas with dense concentrations of factories, as analyzed above. Therefore, in addition to strengthening source control, relocating factories from the vicinity of water sources is also an important measure to protect water sources.

## 4. Conclusions

Based on the calculations of the WQI, the overall assessment of water quality in the study area was deemed good, with relatively average conditions observed throughout the three periods analyzed. Eutrophication of water sources in Huaihe River Basin was severe, with 67.8% of the water sources classified as mesotrophic and 32.2% classified as eutrophic. Water quality and eutrophication levels in the southern mountainous regions were better than those in the densely populated northern areas. TN was the most significant indicator contributing to water body eutrophication.

In terms of health risks, the carcinogenic risk was greater in adults than in children, whereas the noncarcinogenic risk was greater in children than in adults. However, the carcinogenic risk for each heavy metal did not exceed the threshold of 1 × 10^−4^. Cr exhibited the highest carcinogenic risk, with more than 99.8% of both adults and children exposed to a risk higher than 1 × 10^−6^. Additionally, the noncarcinogenic risk posed by the metals did not exceed the threshold of 1, except for Pb.

The results of single factor detection revealed that precipitation played a significant role in controlling As and Pb, with explanatory powers of 0.362 and 0.361, respectively. Additionally, DEM was found to be the dominant factor for Cr, with an explanatory power of 0.457. These three heavy metals were influenced mainly by natural factors but were also indirectly impacted by human factors. On the other hand, the dominant factor for Cd and Hg was GDP, indicating the direct influence of anthropogenic factors.

Furthermore, the results of interaction detection demonstrated that TN, TP, and the other heavy metals were influenced primarily by interactions between natural and anthropogenic factors, except for Hg, which was influenced predominantly by two anthropogenic factors. Compared to single factor analysis, the explanatory power of anthropogenic factors for heavy metals significantly improved when accounting for the indirect influence of natural factors. For example, the explanatory power of Precipitation and Road Network Density on As was 0.362 and 0.189, respectively, whereas their interaction had an explanatory power as high as 0.673. Correlation analysis and principal component analysis also indicated differences between Hg and other heavy metal sources, with areas of higher Hg concentrations typically found near densely populated urban areas. Additionally, TN was strongly influenced by fertilizer application; As was influenced mainly by agricultural activities and transportation; and Cd, Cr, and Pb were influenced mainly by industrial production, particularly by local textile industries such as knitting and clothing factories.

## Data Availability

The datasets used and/or analysed during the current study are available from the corresponding author on reasonable request.

## Supplementary Materials

The following supporting information can be downloaded at: https://www.mdpi.com/article/10.3390/toxics12070517/s1, Figure S1: WQI water quality index during wet, dry, and normal seasons, Figure S2: Calculation results of WQI values in different periods, Figure S3: Calculation of trophic state EI values; Figure S4: Correlation analysis between EI and various factors; Figure S5: Correlation analysis of heavy metals; Table S1: WQI water quality classification, Table S2: EI value of standards at all levels, Table S3: SF and RfD values of each factor, Table S4: Exposure factors and values used in health risk assessment model to evaluate exposure risks with Mote Carlo simulator, Table S5: Concentration statistics of various indicators in drinking water source areas, Table S6: Carcinogenic risk values of various heavy metals, Table S7: Non carcinogenic risk values for various heavy metals, Table S8: The first three terms of factor interaction and their explanatory power q values. References [41,43,49,51,52] are cited in the supplementary materials.
