# Supplementary material for: Quantitative Effects of Anthropogenic and Natural Factors on Heavy Metals Pollution and Spatial Distribution in Surface Drinking Water Sources in the Upper Huaihe River Basin in China
Source: Toxics. 2024 Jul 18;12(7):517. doi: 10.3390/toxics12070517 (PMC11280819; doi:10.3390/toxics12070517)
Supplement: Supplementary file 1 [file toxics-12-00517-s001.zip › toxics-3109642-supplementary.pdf]

Supporting information

Quantitative effects of anthropogenic and natural factors on heavy metals pollution and spatial distribution in surface drinking water sources in the upper Huaihe River Basin in China

Tong Liu[1], Mingya Wang[1], Chunhui Zhang[1], Shili Yang[1], Fan Zhang[1], Luhao Jia[1], Wanqi Ma[1], Shaobo Sui[1],Qingwei Liu[1], Mingshi Wang[1, *]

[1] *College of Resource and Environment, Henan Polytechnic University, Jiaozuo, 454003, China;*

* Corresponding author.

E-mail address: mingshiwang@hpu.edu.cn (M. Wang).

8 Tables 5 Fig

Table S1 WQI water quality classification.

Table S2 EI value of standards at all levels.

Table S3 SF and RfD values of each factor.

Table S4 Exposure factors and values used in health risk assessment model to evaluate exposure risks with Mote Carlo simulator.

Table S5 Concentration statistics of various indicators in drinking water source areas.

Fig S1 WQI water quality index during wet, dry, and normal seasons.

Fig S2 Calculation results of WQI values in different periods.

Fig S3 Calculation of trophic state EI values.

Fig S4 Correlation analysis between EI and various factors.

Table S6 Carcinogenic risk values of various heavy metals.

Table S7 Non carcinogenic risk values for various heavy metals.

Fig S5 Correlation analysis of heavy metals.

Table S8 The first three terms of factor interaction and their explanatory power q values.

Table S1 WQI water quality classification[a]

Range	WQI＜25	25≤WQI≤70	50＜WQI≤70	70＜WQI≤90	WQI＞90
Water quality level	very good	good	medium	poor	very poor

[a]form: (Zhu et al.2021)

Table S2 EI value of standards at all levels[b]

Level	EI
Level 1 (oligotrophic)	20
Level 2 (medium nutrition)	39.42
Level 3 (eutrophic)	61.29
Level 4 (heavy eutrophication)	76.28
Level 5 (extremely nutritious)	99.77

[b]from: (Li et al.2010)

Table S3 SF and RfD values of each factor[c]

	As	Hg	Cd	Cr^{6+}	Pb
SF	1.5		0.5	0.5	0.0085
RfD	0.0003	0.0003	0.0005	0.003	0.0014

[c]from: (Zhang et al.2022)

Table S4 Exposure factors and values used in health risk assessment model to evaluate exposure risks with Mote Carlo simulator

Parameters	Unit	Probabilistic Distribution	Children	Adult	References
C	mg/L	Normal	Measured	Measured	This study
IR (Intake rate)	L/day	Normal	50th :0.6,<br>95th :1.3	50th :1.5,<br>95th :3.7	(Shi et al.2022)[d]
EF (Exposure frequency)	Day/year	Triangular	350 (180,365)	350 (180,365)	(Shi et al.2022)[d]
ED (Exposure duration)	year	Point	6	30	(USEPA,2011)
BW (Average body weight)	kg	Lognormal/Normal	(37.0,2.98)	(60.3,3.46)	(Wang et al.2022)[e]
AT (Average time of exposure)	day	Point	2190 (non-carcinogenic)<br>25550 (carcinogenic)	10950 (non-carcinogenic)<br>25550 (carcinogenic)	(Wang et al.2022)[e]

Table S5 Concentration statistics of various indicators in drinking water source areas

Index	Minimum value (mg/L)	Maximum value (mg/L)	Average value (mg/L)	Median (mg/L)	Standard deviation (mg/L)	Coefficient of variation (%)
pH	6.88	8.90	7.70	7.67	0.46	6.01
DO	5.00	12.2	8.14	8.20	1.56	19.19
COD_{MN}	0.70	5.90	3.32	3.30	1.19	35.85
BOD_5	0.50	3.90	2.51	2.70	0.86	34.40
NH_3-N	0.03	0.85	0.24	0.21	0.14	58.89
TP	0.01	0.19	0.04	0.03	0.02	65.86
TN	0.06	2.5	0.72	0.71	0.28	39.40
As	0.0003	0.003	0.00032	0.0003	0.0003	69.25
Hg	0.00004	0.0001	0.000045	0.00004	0.000015	33.99
Cd	0.0001	0.004	0.0005	0.0005	0.0005	90.99
Cr^{6+}	0.004	0.04	0.005	0.004	0.0039	71.99
Pb	0.00009	0.02	0.0045	0.0025	0.0043	96.15
NO_3-N	0.004	3.08	0.28	0.21	0.31	112.61

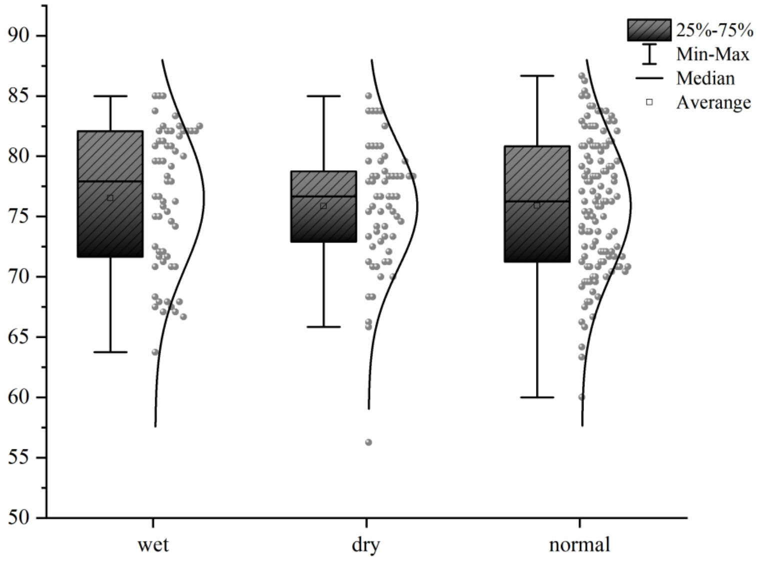

Figure S1 WQI water quality index during wet, dry, and normal seasons

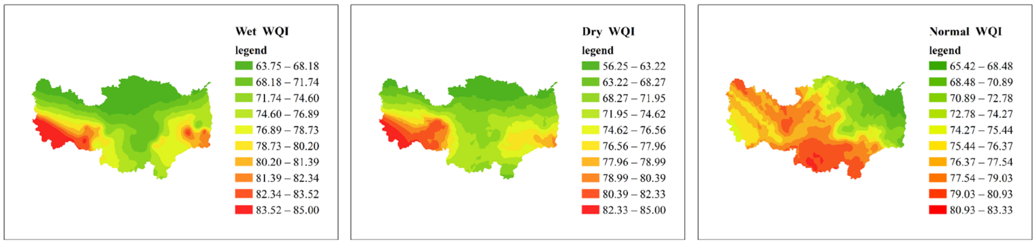

Figure S2 Calculation results of WQI values in different periods

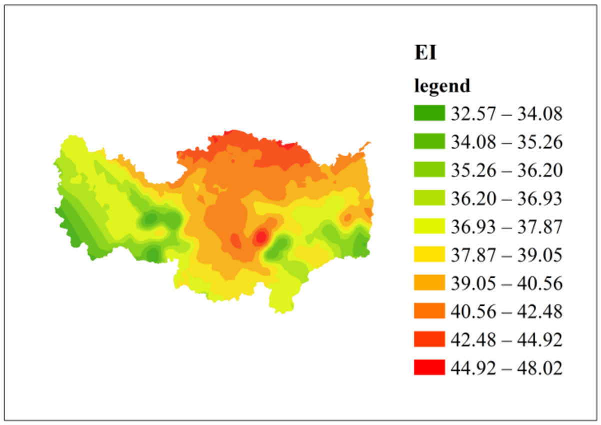

Figure S3 Calculation of trophic state EI values

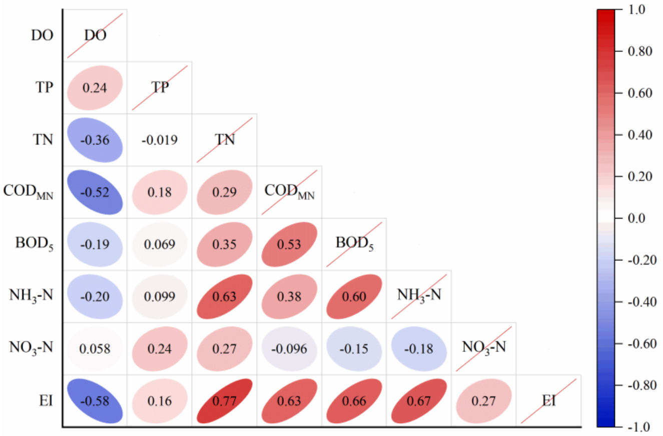

Figure S4 Correlation analysis between EI and various factors

Table S6 Carcinogenic risk values of various heavy metals

		Average value	Average Standard Error	Minimum Value	Maximum Value	Median
As	Children	1.94E-06	9.92E-09	8.63E-08	9.92E-06	1.76E-06
	Adult	7.98E-06	3.44E-08	1.36E-06	3.91E-05	7.36E-06
Cd	Children	7.75E-07	8.67E-09	3.20E-12	1.37E-05	4.94E-07
	Adult	3.17E-06	3.28E-08	9.98E-12	3.58E-05	2.14E-06
Cr^{6+}	Children	6.74E-06	3.97E-08	2.61E-09	3.68E-05	6.24E-06
	Adult	2.76E-05	1.41E-07	1.14E-08	9.48E-05	2.67E-05
Pb	Children	1.07E-07	1.51E-09	1.12E-09	3.61E-06	6.09E-08
	Adult	4.42E-07	6.08E-09	4.43E-09	1.35E-05	2.59E-07

Table S7 Non carcinogenic risk values for various heavy metals

		Average value	Average Standard Error	Minimum Value	Maximum Value	Median
As	Children	5.04E-02	2.57E-04	2.24E-03	2.57E-01	4.55E-02
	Adult	4.14E-02	1.78E-04	7.05E-03	2.03E-01	3.82E-02
Hg	Children	4.93E-03	1.85E-05	2.28E-04	1.62E-02	4.72E-03
	Adult	4.04E-03	1.10E-05	1.56E-03	9.83E-03	3.89E-03
Cd	Children	3.62E-02	4.05E-04	1.50E-07	6.37E-01	2.31E-02
	Adult	2.96E-02	3.06E-04	9.32E-08	3.34E-01	2.00E-02
Cr^{6+}	Children	5.24E-02	3.09E-04	2.03E-05	2.86E-01	4.86E-02
	Adult	4.29E-02	2.19E-04	1.78E-05	1.47E-01	4.15E-02
Pb	Children	1.05E-01	1.48E-03	1.10E-03	3.54E+00	5.97E-02
	Adult	8.66E-02	4.72E-03	8.69E-04	2.64E+00	5.09E-02

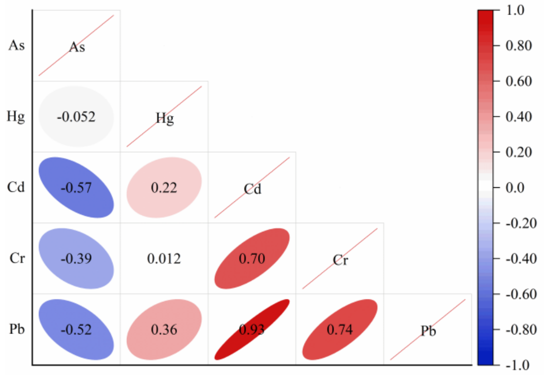

Figure S5 Correlation analysis of heavy metals

Table S8 The first three terms of factor interaction and their explanatory power q values

	Leading factor	interaction1	q value	interaction2	q value	interaction3	q value
TN	Precipitation	$X_5∩X_9$	0.543	$X_3∩X_5$	0.504	$X_5∩X_8$	0.499
TP	GDP	$X_5∩X_8$	0.480	$X_2∩X_7$	0.478	$X_5∩X_7$	0.461
As	Precipitation	$X_2∩X_7$	0.678	$X_3∩X_7$	0.673	$X_1∩X_7$	0.647
Hg	GDP	$X_7∩X_8$	0.763	$X_7∩X_9$	0.607	$X_1∩X_8$	0.567
Cd	GDP	$X_3∩X_7$	0.616	$X_7∩X_8$	0.594	$X_3∩X_8$	0.553
Cr	DEM	$X_1∩X_3$	0.786	$X_1∩X_8$	0.735	$X_1∩X_2$	0.701
Pb	Precipitation	$X_3∩X_8$	0.625	$X_1∩X_7$	0.614	$X_1∩X_3$	0.530

($X_{1\text{-}9}$ represents DEM, NDVI, Precipitation, Temperature, Soil Type, Land Use, Road Network Density, GDP, Population Density)

Reference

Changjun, Z., Fangxing, Z., Budong, L., Shiyan, W., Wenlong, H., 2021. Water quality evaluation of daheiting reservoir based on principal component analysis and wqi_{min} (in chinese). Journal of Henan Normal University(Natural Science Edition) 49 (03), 52-58. https://doi.org/10.16366/j.cnki.1000-2367.2021.03.008.

Zuoyong, L., Jiayang, W., Chun, G., 2010. A universal index formula for eutrophic evaluation using a logarithmic power function (in chinese). Acta Scientiae Circumstantiae 30 (03), 664-672. https://doi.org/10.13671/j.hjkxxb.2010.03.025.

Zhang, L., Tan, X., Chen, H., Liu, Y., Cui, Z., 2022. Effects of agriculture and animal husbandry on heavy metal contamination in the aquatic environment and human health in huangshui river basin. Water (Basel) 14 (4). https://doi.org/10.3390/w14040549.

Shi, H., Zeng, M., Peng, H., Huang, C., Sun, H., Hou, Q., Pi, P., 2022. Health risk assessment of heavy metals in groundwater of hainan island using the monte carlo simulation coupled with the apcs / mlr model. International Journal of Environmental Research and Public Health 19 (13). https://doi.org/10.3390/ijerph19137827.

Wang, W., Chen, C., Liu, D., Wang, M., Han, Q., Zhang, X., Feng, X., Sun, A., Mao, P., Xiong, Q., Zhang, C., 2022. Health risk assessment of pm2.5 heavy metals in county units of northern china based on monte carlo simulation and apcs-mlr. Sci Total Environ 843. https://doi.org/10.1016/j.scitotenv.2022.156777.
